# Adjunctive Maneuvers for Glottic Entry During Pediatric Nasotracheal Intubation: A Prospective Observational Cohort Study

**DOI:** 10.3390/jcm15093455

**Published:** 2026-05-01

**Authors:** Yiru Tong, Jinghui Du, Hengjiang Guo, Rong Wei, Wei Liu, Henry Liu

**Affiliations:** 1 Department of Anesthesiology, Shanghai Children’s Hospital, School of Medicine, Shanghai Jiao Tong University, No. 355 Luding Road, Putuo District, Shanghai 200062, China; 2 Department of Anesthesiology, Bozhou People’s Hospital, Bozhou 236800, China; 3 Department of Anesthesiology and Critical Care, Perelman School of Medicine, University of Pennsylvania, Philadelphia, PA 19104, USA

**Keywords:** nasotracheal intubation, pediatric airway, cuff inflation, Magill forceps, dental surgery

## Abstract

**Background/Objectives:** Pediatric nasotracheal intubation may remain difficult at the stage of glottic alignment and laryngeal entry even after passage through the nasal cavity. We aimed to describe the distribution of adjunctive maneuvers required for glottic entry during pediatric nasotracheal intubation and to explore factors associated with their use. **Methods:** We conducted a prospective observational cohort study of 54 children undergoing dental surgery requiring nasotracheal intubation. Glottic-entry maneuvers were categorized as direct passage, cuff inflation assistance, or Magill forceps assistance. Baseline characteristics, perioperative variables, tube size selection, tube downsizing, and bleeding grade were compared among groups. Univariable and multivariable logistic regression analyses were performed for the primary outcome, defined as the need for any adjunctive maneuver. **Results:** Glottic entry was achieved by direct passage in 34 patients, required cuff inflation assistance in 15, and required Magill forceps assistance in 5. Baseline characteristics were broadly similar across groups, although sex distribution, body weight, and preoperative respiratory rate differed significantly. Anesthesia duration, surgery duration, bleeding grade, initial tube size, tube downsizing, and final tube size did not differ significantly among groups. In univariable logistic regression, female sex showed a borderline association with the need for any adjunctive maneuver, whereas age and initial tube size were not significant. In multivariable analysis, no stable independent predictor was identified. **Conclusions:** Most children achieved glottic entry by direct passage, whereas a minority required cuff inflation assistance or Magill forceps assistance. These prospective data provide a practical stepwise description of glottic-entry assistance during pediatric nasotracheal intubation.

## 1. Introduction

Pediatric nasotracheal intubation is commonly used for dental and maxillofacial procedures because it secures the airway while preserving access to the oral cavity [1,2]. However, advancing the tube into the trachea may be technically challenging in children [1,2].

Most discussions of difficulty during pediatric nasotracheal intubation focus on nasal passage, tube selection and depth, and upper-airway complications such as mucosal trauma and epistaxis [1,3,4,5]. In clinical practice, difficulty may persist even after the tube has passed the nasal cavity, particularly at the stage of glottic alignment and laryngeal entry. At this point, direct passage refers to advancement of the nasotracheal tube into the glottis without an auxiliary maneuver; cuff inflation assistance refers to transient inflation of the tube cuff to redirect the tube tip toward the laryngeal inlet; and Magill forceps assistance refers to laryngoscopic grasping and guidance of the tube tip into the glottis [6,7,8,9]. For dental practitioners and pediatric anesthesiologists, this stage is clinically important because repeated attempts to align the tube with the laryngeal inlet may prolong intubation and increase mucosal trauma, bleeding, and procedural difficulty in a small pediatric airway [1,3,4,5].

Although these maneuvers are familiar to airway practitioners, their distribution and associated clinical features have not been well characterized in prospective pediatric cohorts. Current pediatric literature has mainly focused on anatomy, tube size and insertion depth, device comparisons, and complications of nasotracheal intubation, whereas prospective data specifically addressing the glottic-entry stage remain limited [1,6,10]. A prospective description of how frequently each maneuver is required in routine pediatric dental anesthesia may therefore help procedural planning, stepwise escalation, and technical training [1,6,7,8,9]. In particular, it remains unclear how often adjunctive maneuvers are required and whether baseline patient characteristics or procedural factors are associated with this need. We hypothesized that direct passage would be feasible in most children and that adjunctive maneuvers would be required in a minority.

We therefore conducted a prospective observational cohort study in children undergoing dental surgery with nasotracheal intubation. The aims of this study were to describe the distribution of direct passage, cuff inflation assistance, and Magill forceps assistance for glottic entry; to compare baseline and perioperative characteristics among these groups; and to explore factors associated with the need for any adjunctive maneuver and, secondarily, the need for Magill forceps assistance.

## 2. Materials and Methods

### 2.1. Study Design and Ethics

This study was a prospective observational cohort analysis of children undergoing dental surgery requiring nasotracheal intubation. The study was approved by the Institutional Review Board of Shanghai Children’s Hospital (Approval No. 2024R011-F01; 30 January 2024). Written informed consent for study participation was obtained from legal guardians before enrollment. Written informed consent for publication was obtained from legal guardians for identifiable images or videos where applicable.

### 2.2. Patients

Eligible participants were children younger than 18 years undergoing dental surgery under general anesthesia with planned nasotracheal intubation and an expected operative duration of more than 1 h. Consecutive eligible patients meeting these criteria were prospectively enrolled in this observational cohort. Because this was an observational study, airway management was performed according to routine clinical practice rather than a protocol-assigned intervention, and the required glottic-entry maneuver was documented prospectively for analysis. Patients were not enrolled if legal guardians declined informed consent, and no additional exclusion criteria beyond the above clinical eligibility requirements were applied during implementation of the study. Baseline demographic and clinical data were collected before anesthesia induction, including sex, age, height, weight, preoperative heart rate, respiratory rate, systolic blood pressure, diastolic blood pressure, and comorbidity status.

### 2.3. Intubation Procedure and Maneuver Classification

Nasotracheal intubation was initially attempted using the standard clinical approach under videolaryngoscopic guidance with a TDC-2 video laryngoscope (product code: 18102224; Zhejiang UE Medical Instrument Co., Ltd., Taizhou, Zhejiang, China). A Parker reinforced tracheal tube with a standard tip (Bell Medical, St. Louis, MO, USA) was used according to routine clinical practice. For the purpose of this study, the glottic-entry stage was defined as the interval after nasal passage of the tube when the tube tip approached the laryngeal inlet under videolaryngoscopic visualization. If the tube tip aligned with and entered the glottis without an auxiliary maneuver, the case was classified as direct passage. If direct passage was not achieved, transient cuff inflation was used to elevate and redirect the tube tip toward the laryngeal inlet; cases successfully completed with this maneuver were classified as cuff inflation assistance. If glottic entry still could not be achieved, Magill forceps were used under laryngoscopic visualization to guide the tube tip into the glottis; these cases were classified as Magill forceps assistance. Advancement to the next maneuver occurred only if the preceding maneuver did not achieve satisfactory alignment or glottic entry. For analysis, each patient was assigned to the highest level of maneuver required to achieve glottic entry.

For regression analyses, two binary outcomes were defined. The primary outcome (arm1) was direct passage versus any adjunctive maneuver. The secondary exploratory outcome (arm2) was direct passage or cuff inflation assistance versus Magill forceps assistance.

### 2.4. Variables Collected

In addition to baseline variables, the following perioperative and intubation-related variables were recorded: anesthesia duration, surgery duration, bleeding grade, initial tube size, tube downsizing, and final tube size.

Mucosal trauma and epistaxis were graded using a prespecified study-specific 0–4 bleeding scale: 0, no bleeding; 1, congestion or minor mucosal injury without visible blood; 2, mild bleeding or oozing without pooling; 3, persistent bleeding requiring repeated suction; and 4, severe bleeding requiring hemostatic intervention.

### 2.5. Outcomes

The primary outcome was the need for any adjunctive maneuver for glottic entry during nasotracheal intubation. The secondary exploratory outcome was the need for Magill forceps assistance.

### 2.6. Statistical Analysis

Continuous variables were summarized as mean ± standard deviation or median [interquartile range], as appropriate, and categorical variables as number (percentage). Comparisons among the three maneuver groups were performed using one-way ANOVA, the Kruskal–Wallis test, Fisher’s exact test, or chi-square testing, as appropriate.

Binary logistic regression was used to assess factors associated with the primary outcome, defined as the need for any adjunctive maneuver for glottic entry during nasotracheal intubation (arm1: 0 = direct passage; 1 = cuff inflation assistance or Magill forceps assistance). Female sex was specified as the main independent variable, and age in months was included as an adjusting covariate in the primary multivariable model. Because the initial tube size was considered procedurally relevant, an exploratory sensitivity model additionally included the initial tube size. Sex was evaluated as an exploratory clinically relevant variable because potential sex-related differences in airway or procedural characteristics might influence glottic-entry difficulty; however, given the sample size, any observed association was interpreted cautiously. No imputation was performed for missing data, and analyses were performed using a complete-case approach. Odds ratios (ORs) with 95% confidence intervals (CIs) and two-sided *p* values were reported.

A secondary exploratory analysis examined factors associated with the need for Magill forceps assistance (arm2: 0 = direct passage or cuff inflation assistance; 1 = Magill forceps assistance). Because only five patients required Magill forceps assistance, this analysis was limited to descriptive comparisons and univariable logistic regression, and no multivariable model was fitted.

A two-sided α level of 0.05 was considered statistically significant. No adjustment was made for multiple comparisons. All analyses were performed using R version 4.3.2 (R Foundation for Statistical Computing, Vienna, Austria).

## 3. Results

### 3.1. Study Population and Maneuver Distribution

A total of 54 children were included in the analysis. Glottic entry was achieved by direct passage in 34 patients, required cuff inflation assistance in 15 patients, and required Magill forceps assistance in 5 patients.

### 3.2. Baseline Characteristics

Baseline characteristics by maneuver group are shown in Table 1. Overall, the three groups were broadly comparable in age, height, preoperative heart rate, systolic blood pressure, diastolic blood pressure, and comorbidity status. However, sex distribution differed significantly across groups (*p* = 0.004), and significant between-group differences were also observed for body weight (*p* = 0.005) and preoperative respiratory rate (*p* = 0.015). The Magill forceps group tended to have a higher body weight than the other groups. Given the small size of the Magill forceps group, differences in sex distribution should be interpreted cautiously.

### 3.3. Perioperative and Intubation-Related Outcomes

Perioperative and intubation-related outcomes are summarized in Table 2. Anesthesia duration and surgery duration did not differ significantly among groups. Similarly, no significant group differences were observed in bleeding grade, initial tube size, tube downsizing, or final tube size. Although the bleeding scale ranged from 0 to 4, only grades 0 to 2 were observed in this cohort.

### 3.4. Primary Regression Analysis: Need for Any Adjunctive Maneuver

Logistic regression results for the primary outcome are summarized in Table 3 (A). In univariable logistic regression, female sex showed a borderline association with the need for any adjunctive maneuver (OR 3.25, 95% CI 1.01–10.98, *p* = 0.051), whereas age was not significantly associated with the outcome (OR 0.97, 95% CI 0.93–1.01, *p* = 0.166). Initial tube size was also not significantly associated with the primary outcome in univariable analysis (OR 0.53, 95% CI 0.10–2.59, *p* = 0.443).

In the primary multivariable logistic regression model including female sex and age, neither variable remained independently associated with the need for any adjunctive maneuver. Female sex retained a borderline trend (OR 2.77, 95% CI 0.82–9.78, *p* = 0.105), whereas age was not significant (OR 0.98, 95% CI 0.93–1.03, *p* = 0.383).

### 3.5. Sensitivity Analysis Including Initial Tube Size

In an exploratory sensitivity model additionally including initial tube size (Table 3 (A)), the overall interpretation of the primary analysis was unchanged. Female sex remained borderline associated with the primary outcome (OR 3.16, 95% CI 0.89–11.99, *p* = 0.079), whereas age (OR 0.95, 95% CI 0.88–1.02, *p* = 0.201) and initial tube size (OR 3.91, 95% CI 0.24–72.47, *p* = 0.342) were not significantly associated with the outcome. The wide confidence interval for initial tube size indicated the imprecision of estimation.

### 3.6. Secondary Exploratory Analysis: Need for Magill Forceps Assistance

Secondary exploratory regression results for the need for Magill forceps assistance are summarized in Table 3 (B). In this analysis, only five patients met the outcome definition. Univariable logistic regression did not identify significant associations for age or initial tube size. The estimate for sex was unstable because no female patient required Magill forceps assistance. No multivariable logistic regression model was fitted because of the small number of events and the instability of the estimates.

## 4. Discussion

This prospective observational study provides a practical description of glottic-entry maneuvers during pediatric nasotracheal intubation in children undergoing dental surgery. Most patients were intubated by direct passage, whereas a smaller proportion required cuff inflation assistance and only a few required Magill forceps assistance. Thus, escalation beyond direct passage appeared necessary in a minority of children in this cohort.

Baseline and perioperative characteristics were generally similar across maneuver groups. Although significant between-group differences were observed in sex distribution, body weight, and preoperative respiratory rate, these findings should be interpreted cautiously, particularly because the Magill forceps group comprised only five patients. In contrast, anesthesia duration, surgery duration, bleeding grade, and tube size-related variables did not differ significantly across groups.

In regression analysis, female sex showed a borderline association with the need for any adjunctive maneuver, whereas age and initial tube size were not significantly associated with the primary outcome. After multivariable adjustment, no independent predictor was identified. Because the observed sex association was only borderline in this relatively small cohort, it should be regarded as exploratory rather than mechanistic. These findings suggest that the requirement for glottic-entry assistance may not be easily predicted by routine baseline variables alone in a small prospective clinical cohort.

Because the initial tube size was procedurally relevant, we further examined it in a sensitivity model. Although this addition did not materially alter the overall conclusions, the estimate for initial tube size was imprecise and accompanied by a wide confidence interval. This likely reflects the limited sample size and event number rather than a robust absence or presence of association.

Our findings fit best with a stepwise technical interpretation rather than a strong predictive model. Current pediatric nasotracheal intubation literature has largely focused on anatomy, tube size and insertion depth, device comparisons, and complications rather than on prospective characterization of glottic-entry maneuvers themselves [1,6,10]. Prior studies have shown that cuff inflation can facilitate advancement of the nasotracheal tube toward the laryngeal inlet and, in some settings, reduce reliance on Magill forceps [7,8,9,11]. In this context, the present cohort adds prospective pediatric data focused specifically on the glottic-entry stage and may help bridge the gap between general technical descriptions and a more structured stepwise approach to pediatric dental nasotracheal intubation.

The secondary exploratory analysis focusing on Magill forceps assistance was constrained by the very small number of events. As a result, no stable multivariable model could be constructed. This underscores that escalation to Magill forceps was uncommon in this cohort and that larger studies would be required to define its predictors reliably.

Clinically, these findings support a stepwise understanding of glottic-entry management during pediatric nasotracheal intubation. In this cohort, direct passage was feasible; when not, cuff inflation may provide a useful intermediate maneuver before resorting to Magill forceps assistance [7,8,9]. Even without strong predictive factors, documenting the frequency and sequence of these maneuvers may help improve procedural anticipation and technical training. From a practical standpoint, the present data may assist pre-intubation preparation, shared anticipation of escalation steps within the airway team, and teaching of a structured progression from direct passage to cuff inflation and finally to Magill forceps assistance when needed. Future studies should evaluate these maneuvers in larger multicenter pediatric cohorts, incorporate anatomical or imaging-based predictors, and assess whether standardized stepwise algorithms can reduce repeated manipulation, intubation time, or airway trauma.

This study has several limitations. First, it was conducted in a relatively small single-center cohort. Second, the number of Magill forceps cases was very small, limiting secondary regression analysis. Third, not all potentially relevant anatomical measurements were available for the full cohort, and these were therefore not incorporated into the primary analyses. Fourth, the observational design precludes causal inference. Finally, the findings are specific to children undergoing dental surgery and may not be generalizable to other patient populations or procedural settings.

## 5. Conclusions

Glottic entry during pediatric nasotracheal intubation was achieved by direct passage in most children, whereas cuff inflation assistance and Magill forceps assistance were required in a minority. No stable independent predictor of adjunctive maneuver requirement was identified, although female sex showed a borderline association with the primary outcome. These prospective data provide a clinically useful description of stepwise glottic-entry assistance during pediatric nasotracheal intubation.

## Figures and Tables

**Table 1:** Baseline characteristics according to glottic-entry maneuver group.

Characteristic	Direct Passage (*n* = 34)	Cuff Inflation (*n* = 15)	Magill Forceps (*n* = 5)	*p* Value
Female sex, *n* (%)	8 (23.5)	10 (66.7)	0 (0.0)	0.004
Age, months	62.79 ± 13.88	56.07 ± 11.63	61.60 ± 16.41	0.282
Height, cm	111.97 ± 8.57	108.13 ± 10.49	115.20 ± 9.26	0.249
Weight, kg	19.0 [18.0–22.0]	18.0 [15.65–18.50]	24.0 [20.5–26.0]	0.005
Preoperative heart rate, beats/min	96.0 [96.0–101.5]	98.0 [96.0–99.0]	99.0 [94.0–99.0]	0.928
Preoperative respiratory rate, breaths/min	22.0 [20.0–23.0]	23.0 [23.0–24.0]	22.0 [20.0–22.0]	0.015
Preoperative systolic blood pressure, mmHg	101.5 [98.0–103.0]	103.0 [96.0–103.5]	101.0 [100.0–105.0]	0.983
Preoperative diastolic blood pressure, mmHg	60.5 [52.25–62.0]	61.0 [60.0–63.0]	65.0 [60.0–65.0]	0.183
Comorbidity present, *n* (%)	8 (23.5)	2 (13.3)	1 (20.0)	0.872

Data are presented as mean ± SD, median [IQR], or n (%), as appropriate. *p* values were calculated using one-way ANOVA, the Kruskal–Wallis test, or Fisher’s exact test, as appropriate.

**Table 2:** Intubation-related and perioperative outcomes according to glottic-entry maneuver group.

Outcome	Direct Passage (*n* = 34)	Cuff Inflation (*n* = 15)	Magill Forceps (*n* = 5)	*p* Value
Anesthesia duration, min	132.41 ± 42.42	114.00 ± 33.68	138.40 ± 33.83	0.275
Surgery duration, min	119.91 ± 41.77	100.80 ± 33.28	125.20 ± 36.72	0.252
Bleeding grade, *n* (%)				0.905
0 (none)	27 (79.4)	10 (66.7)	4 (80.0)	
1	2 (5.9)	1 (6.7)	0 (0.0)	
2	5 (14.7)	4 (26.7)	1 (20.0)	
Initial tube size, mm	4.43 ± 0.35	4.30 ± 0.32	4.50 ± 0.50	0.455
Tube downsizing, *n* (%)	2 (5.9)	1 (6.7)	1 (20.0)	0.693
Final tube size, mm	4.40 ± 0.34	4.23 ± 0.26	4.40 ± 0.55	0.342

Data are presented as mean ± SD or n (%), as appropriate. Bleeding grades 0–2 are shown because grades 3 and 4 were not observed in this cohort. p values were calculated using one-way ANOVA, the Kruskal–Wallis test, or chi-square/Fisher’s exact testing, as appropriate.

**Table 3:** Logistic regression analyses for the need for adjunctive maneuver and Magill forceps assistance. (A) Primary outcome: need for any adjunctive maneuver (arm1). (B) Secondary exploratory outcome: need for Magill forceps assistance (arm2).

(A)			
Variable	OR	95% CI	*p* Value
Univariable analysis			
Female sex	3.25	1.01–10.98	0.051
Age, months	0.97	0.93–1.01	0.166
Initial tube size, mm	0.53	0.10–2.59	0.443
Primary multivariable model			
Female sex	2.77	0.82–9.78	0.105
Age, months	0.98	0.93–1.03	0.383
Sensitivity model			
Female sex	3.16	0.89–11.99	0.079
Age, months	0.95	0.88–1.02	0.201
Initial tube size, mm	3.91	0.24–72.47	0.342
(B)			
Variable	OR	95% CI	*p* Value
Female sex	Unstable estimate *	—	—
Age, months	1.00	0.94–1.08	0.891
Initial tube size, mm	2.43	0.17–36.19	0.501

arm1 represents the primary outcome, any adjunctive maneuver versus direct passage. arm2 represents the secondary exploratory outcome, Magill forceps assistance versus direct passage or cuff inflation assistance. * The estimate for sex in arm2 was unstable because no female patient required Magill forceps assistance.

## Data Availability

The data presented in this study are available on reasonable request from the corresponding author.

## References

[1] Kim J., Jeon S. (2024). Nasotracheal intubation in pediatrics: A narrative review. J. Dent. Anesth. Pain Med..

[2] Ozkan A.S., Akbas S. (2018). Nasotracheal intubation in children for outpatient dental surgery: Is fiberoptic bronchoscopy useful?. Niger. J. Clin. Pract..

[3] El-Seify Z.A., Khattab A.M., Shaaban A.A., Metwalli O.S., Hassan H.E., Ajjoub L.F. (2010). Xylometazoline pretreatment reduces nasotracheal intubation-related epistaxis in paediatric dental surgery. Br. J. Anaesth..

[4] Watt S., Pickhardt D., Lerman J., Armstrong J., Creighton P.R., Feldman L. (2007). Telescoping tracheal tubes into catheters minimizes epistaxis during nasotracheal intubation in children. Anesthesiology.

[5] Yamamoto T., Flenner M., Schindler E. (2019). Complications associated with nasotracheal intubation and proposal of simple countermeasure. Anaesthesiol. Intensive Ther..

[6] Kim H.-J., Kim J.-T., Kim H.-S., Kim C.-S., Kim S.-D. (2011). A comparison of GlideScope^®^ videolaryngoscopy and direct laryngoscopy for nasotracheal intubation in children. Paediatr. Anaesth..

[7] Kumar R., Gupta E., Kumar S., Sharma K.R. (2013). Cuff inflation-supplemented laryngoscope-guided nasal intubation: A comparison of three endotracheal tubes. Anesth. Analg..

[8] Lin C.-H., Tseng K.-Y., Su M.-P., Chuang W.-M., Hu P.-Y., Cheng K.-I. (2022). Cuff inflation technique is better than Magill forceps technique to facilitate nasotracheal intubation guiding by GlideScope^®^ video laryngoscope. Kaohsiung J. Med. Sci..

[9] Chung Y.-T., Sun M.-S., Wu H.-S. (2003). Blind nasotracheal intubation is facilitated by neutral head position and endotracheal tube cuff inflation in spontaneously breathing patients. Can. J. Anesth..

[10] Chou C.-H., Tsai C.-L., Lin K.-L., Wu S.-C., Chiang M.-H., Huang H.-W., Hung K.-C. (2023). A new formula to predict the size and insertion depth of cuffed nasotracheal tube in children receiving dental surgery: A retrospective study. Sci. Rep..

[11] Ono K., Goto T., Nakai D., Ueki S., Takenaka S., Moriya T. (2014). Incidence and predictors of difficult nasotracheal intubation with airway scope. J. Anesth..

